# The pathophysiology of leg cramping during dialysis and the use of carnitine in its treatment

**DOI:** 10.14814/phy2.15114

**Published:** 2021-11-11

**Authors:** Akira Takahashi

**Affiliations:** ^1^ Tesseikai Neurosurgical Hospital Shijonawate Japan

**Keywords:** acyl coenzyme A, ATP, carnitine, coenzyme Q10, contraction alkalosis, leg cramping, muscle cramp

## Abstract

Leg cramping is a common side effect of hemodialysis, and this is frequently treated by the administration of carnitine, but this is not effective in every patient. Alkalosis is a key component of the etiology of leg cramping during hemodialysis sessions. This is mediated through the binding of calcium ions to serum albumin, which causes hypocalcemia, and an increase in the release of calcium ions from the sarcoplasmic reticulum. Normally the calcium pump on the sarcoplasmic reticulum consumes ATP and quickly reuptakes the released calcium ions, which rapidly stops excessive muscle contractions. Thus, carnitine deficiency results in prolonged muscle contraction because of ATP depletion. However, during ATP production, carnitine is only involved up to the stage of acyl‐CoA transport into mitochondria, and for the efficient generation of ATP, the subsequent metabolism of acyl‐CoA is also important. For example, β‐oxidation and the tricarboxylic acid cycle may be affected by a deficiency of water‐soluble vitamins and the electron transport chain requires coenzyme Q10, but statins inhibit its production. The resulting accumulation of excess long‐chain acyl‐CoA in mitochondria inhibits enzymes involved in energy production. Thus, carnitine administration may be used more effectively if clinicians are aware of its specific physiologic roles.

## Graphical abstract

A key component of the etiology of leg cramping during hemodialysis sessions is alkalosis, which induces hypocalcemia and the release of calcium ions from the sarcoplasmic reticulum. Alkalosis is caused by not only dialysate but also contraction alkalosis due to fluid loss. The ATP provided by the correct use of carnitine supplementation ensures the reabsorption of calcium into the sarcoplasmic reticulum, which rapidly stops the excessive muscle contractions (Figure 3). The routine use of carnitine in patients undergoing hemodialysis is now becoming established in Japan.

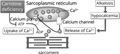

## 1 INTRODUCTION

According to previous reports, muscle spasms occur in 33%–86% of patients that undergo hemodialysis (Canzanello & Burkart, 1992; Kobrin & Berns, 2007; Punj et al., 2020). In patients undergoing peritoneal dialysis, the prevalence of muscle spasms may be much higher (Figueiredo et al., 2012) or similar to that in patients undergoing hemodialysis (Weisbord et al., 2005). A study of dialysis‐related muscle cramping by Punj et al. (2020) showed that 79% of the participants (117 of 149) had experienced cramping at least once during dialysis, and 73% (85 of 117) of them reported that this occurred during the last hour of the session.

The pathogenesis of this leg cramping during the latter half of hemodialysis sessions has been thought to be the result of hypotension caused by the excessive removal of water during dialysis (Kaplan et al., 1992; Santoro et al., 1998) or of the dialysate tending to cause acidosis (Kasserra et al., 1994; Wagner et al., 1983). However, the etiology has not been fully descried.

In this review article, I first describe the physiological role of key nutrients, and especially that of carnitine (Hatanaka et al., 2019; Shimizu et al., 2019), then discuss the pathophysiology of this leg cramping with reference to the key defect of contraction alkalosis (Garella et al., 1975), which refers to the increase in blood pH that occurs as a result of fluid loss (volume contraction). I then discuss the significance of the deficiency of carnitine in the onset of muscular symptoms in patients undergoing hemodialysis, and finally, I discuss preventive and therapeutic strategies for this phenomenon, with a specific focus on the clinical utility of carnitine supplementation.

## 2 THE PHYSIOLOGIC ROLE OF CARNITINE

The muscle symptoms that arise during hemodialysis are thought to be caused by defects in fatty acid transport, which can be directly related to carnitine deficiency (Sakurauchi et al., 1998). Therefore, it is important to understand the physiologic role of carnitine in muscle.

### 2.1 Carnitine and energy production

**FIGURE 1:**
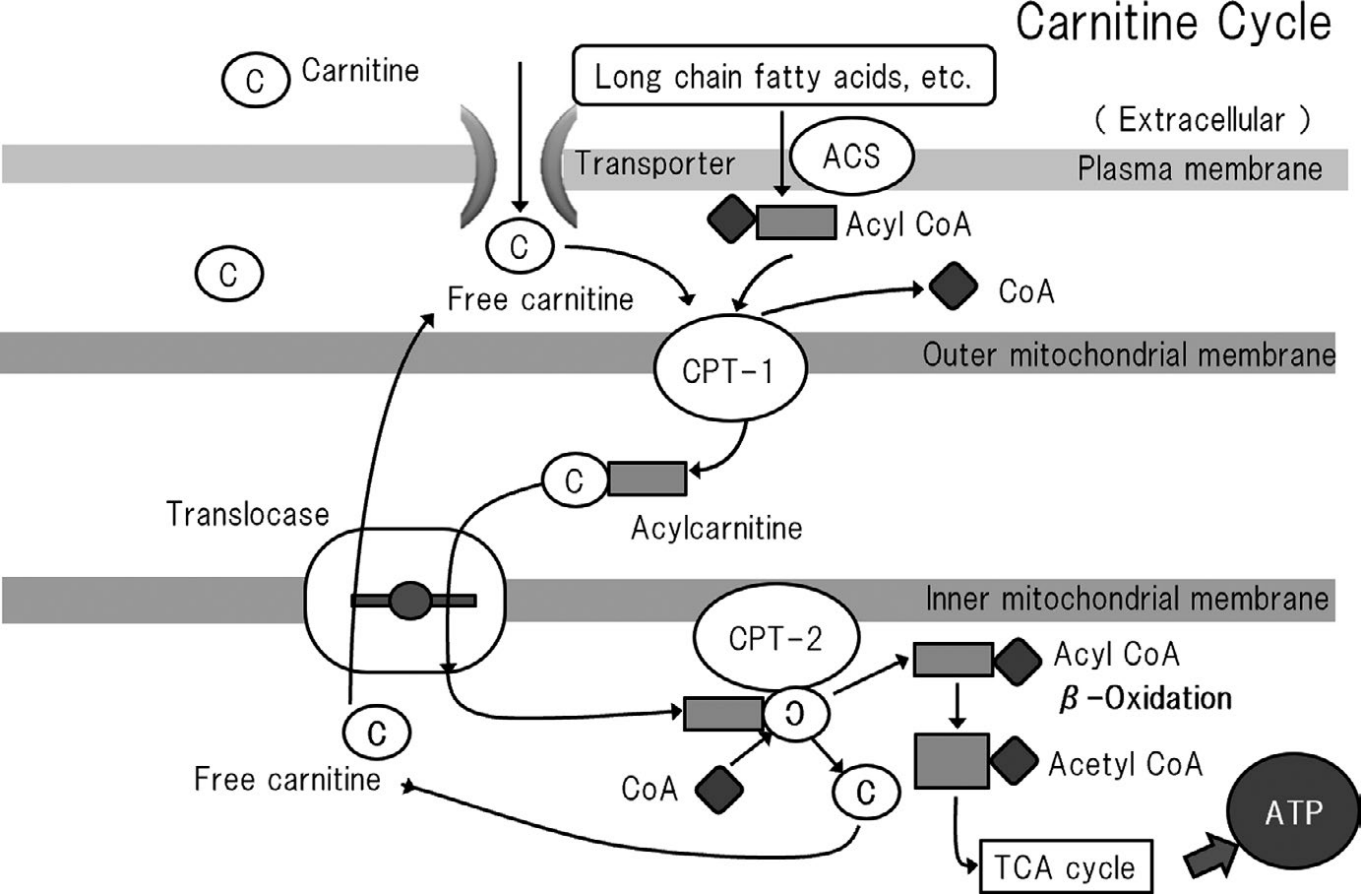
The carnitine shuttle system. Carnitine is necessary for the transport of fatty acids into mitochondria, which is accomplished as part of the long‐chain fatty acid transport system, referred to as the carnitine circuit or carnitine shuttle, which also comprises several enzymes in the mitochondrial membrane, and plays an important role in energy production from fatty acids. Organic ion/carnitine transporter 2 (OCTN2) is expressed in cell membranes and has the effect of concentrating carnitine to 20–50 times its extracellular concentration. Free fatty acids are converted to acyl‐CoAs by long‐chain acyl‐CoA synthetase (ACS) on the outer mitochondrial membrane and then transported into the space between the outer and inner mitochondrial membranes. Here, a reaction between acyl‐CoA and carnitine occurs that is catalyzed by carnitine palmitoyltransferase I (CPT‐1) on the inside of the outer mitochondrial membrane, and the acylcarnitine generated is transported to the inner mitochondrial matrix by carnitine acylcarnitine translocase (CACT) on the inner mitochondrial membrane. The acylcarnitine is then broken down to liberate carnitine and long‐chain fatty acids by carnitine palmitoyltransferase 2 (CPT‐2) that is expressed inside the inner mitochondrial membrane, and the released fatty acids undergo β‐oxidation in the mitochondrial matrix to generate energy. Free carnitine then returns to the intermembrane space *via* CACT. Thus, free carnitine and acylcarnitine are transported in opposite directions, whether the movement of acylcarnitine is inward or outward. However, this circuit cannot operate if there is an absolute deficiency of free carnitine or if there is a shortage of free carnitine relative to the amount of acyl‐CoA (carnitine insufficiency) (Angelini et al., 1992). C, carnitine; ACS, acyl‐CoA synthetase; CPT, carnitine palmitoyltransferase, CoA, coenzyme A

Carnitine is required for the transport of fatty acids into (Angelini et al., 1992) and out of (Steiber et al., 2004; Zammit et al., 2009) mitochondria and for the stabilization of cell membranes (Bonomini et al., 2011). Specifically, it facilitates the transport of long‐chain fatty acids between the cytosol and mitochondria. Initially, long‐chain fatty acids are converted to acyl‐CoA by acyl‐CoA synthetase (ACS). The acyl‐CoA generated is then linked to carnitine by carnitine mitochondrial palmitoyltransferase (CPT), forming acylcarnitine. A translocase is responsible for the passive transport of carnitine and acylcarnitine, returning one molecule of carnitine from the mitochondrial matrix to the intermembrane space at the same time as one molecule of fatty acyl‐carnitine moves into the matrix. The acylcarnitine that is transported across to the inner mitochondrial membrane is reconverted to acyl‐CoA by CPT. In this way, carnitine acts as a shuttle for fatty acids (Angelini et al., 1992) (Figure 1).

Acyl‐CoA that enters the mitochondria is converted to acetyl‐CoA *via* β‐oxidation, and the acetyl‐CoA molecules generated then enter the tricarboxylic acid (TCA) cycle. One molecule of acetyl‐CoA is used to generate three molecules of NADH, one molecule of FADH_2_, and one molecule of GTP during each revolution of the TCA cycle. These NADH_2_ and FADH_2_ molecules are then transferred to the electron transport chain, where they are used to produce ATP (Stanley, 2004).

During glycolysis, only two ATP molecules are generated from one molecule of glucose, but many more are generated from fatty acid molecules. For example, a net total of 106 ATP molecules are generated in mitochondria from one 16‐carbon molecule of palmitic acid, as a result of the generation of eight molecules of acetyl‐CoA and seven molecules of NADH and FADH_2_ by β‐oxidation; and 31 NADH, 15 FADH_2_, and 8 GTP molecules by the TCA cycle, at the expense of two ATP molecules that are required to convert one molecule of palmitic acid to palmitoyl‐CoA (Stipanuk, 2018). Thus, fatty acids represent rich sources of energy. However, carnitine deficiency results in a lack of supply of long‐chain fatty acids for β‐oxidation, and therefore lower acetyl‐CoA production, which reduces the production of NADH_2_ and FADH_2_ in the TCA cycle and ATP production by the electron transport chain.

### 2.2 Carnitine regulates the supply of acyl groups to the mitochondria

**FIGURE 2:**
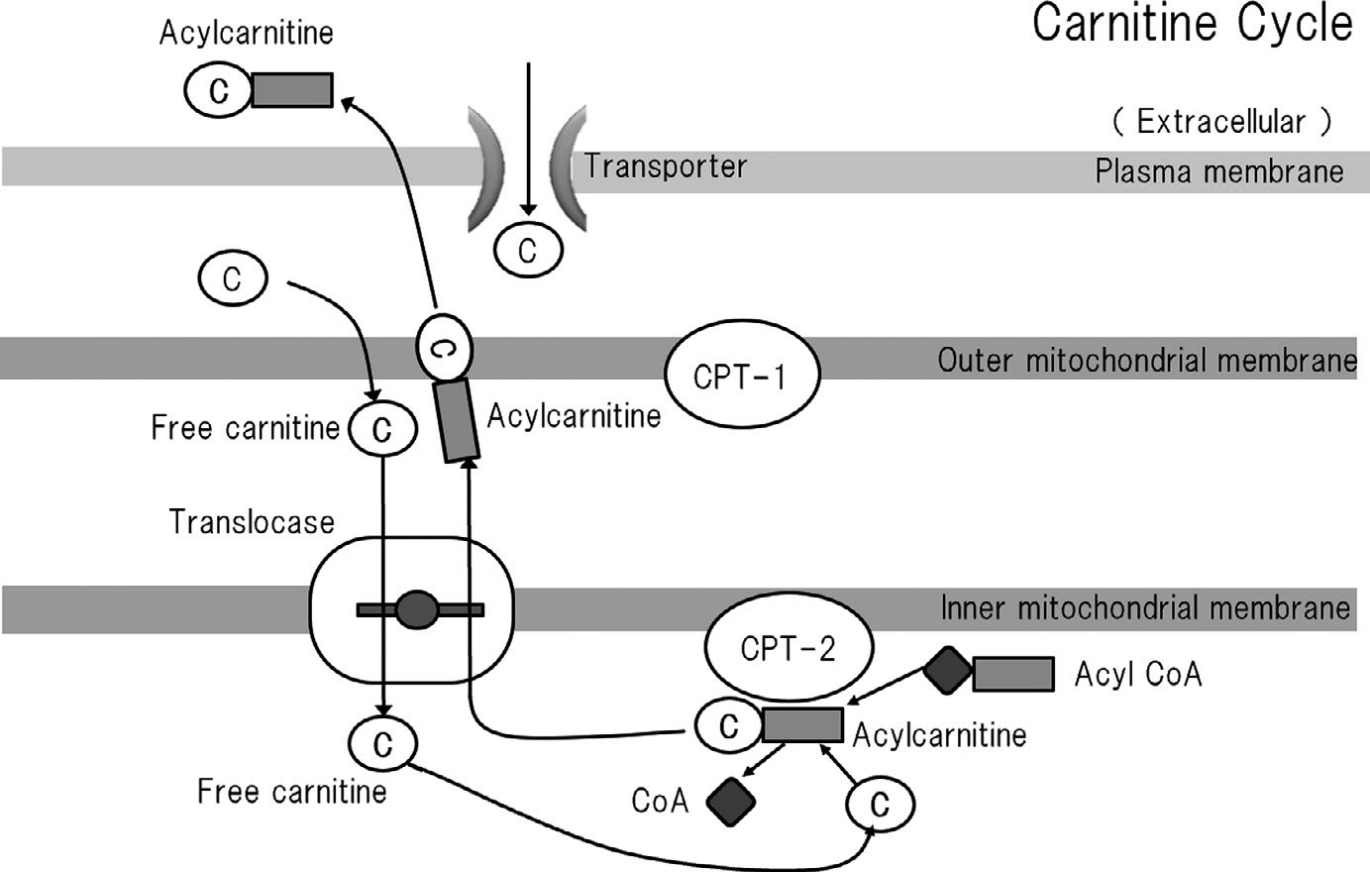
Carnitine is necessary for the export of fatty acids from mitochondria. Free carnitine binds to the acyl group of acyl‐CoA to form acylcarnitine, and is excreted extracellularly. Acyl compounds are metabolic intermediates, but are cytotoxic when they accumulate in individuals with organic acid metabolism disorders. These cytotoxic effects are mediated through the inhibition of various mitochondrial enzymes. Under normal circumstances, free carnitine is used to remove excess acyl‐CoA from cells as the carnitine ester acylcarnitine, which is excreted in the urine. Therefore, free carnitine represents an endogenous means of preventing the deleterious effects of acyl compounds (Stumpf et al., 1985). ACS, acyl‐CoA synthetase; CPT, carnitine palmitoyltransferase

Carnitine regulates the ratio of acyl‐CoA to free CoA in mitochondria (Brass & Hoppel, 1980; Friolet et al., 1994) (Figure 2). The following reversible reaction is used to regulate the cellular free CoA concentration, in which carnitine is used as a buffer (Stephens et al., 2007):

Carnitine+Acyl‐CoA⇔Acyl‐Carnitine+CoA.

Carnitine insufficiency has been shown to result in the accumulation of excess acyl‐CoA in the mitochondria of rat myocardium and liver, which alters mitochondrial membrane permeability, leading to cytochrome C release, caspase 3 activation, and ceramide accumulation, which result in apoptosis (Furuno et al., 2001; Oyanagi et al., 2015). In addition, the accumulation of acyl‐CoA in mitochondria inhibits many enzymes that are involved in energy production, such as in the TCA cycle (Stumpf et al., 1985) (Table 1). Acyl‐CoA accumulation also destabilizes cell membranes in the myocardium, causing arrhythmia (Russell et al., 1981), and palmitoyl‐CoA has been shown to cause cardiomyocyte apoptosis (Sparagna et al., 2000).

**TABLE 1:** Enzymes inhibited by acyl‐CoA compounds

Enzyme	Activity
Acetyl‐CoA carboxylase	Regulation of fatty acid synthesis and β‐oxidation
Adenine nucleotide translocase	Transportation of ATP out of mitochondria
Citrate synthetase	Involved in the TCA cycle
Glutamate dehydrogenase	Involved in the synthesis of urea
Lipase	Catalyzes the hydrolysis of lipids
Malate dehydrogenase	Involved in pyruvate metabolism
N‐acetyl glutamate synthetase	Involved in the urea cycle
3‐Oxoacyl‐CoA thiolase	Involved in fatty acid synthesis
Pyruvate dehydrogenase complex	Involved in the TCA cycle
Pyruvate dehydrogenase kinase	Involved in the TCA cycle
Pyruvate carboxylase	Involved in the TCA cycle
Succinate‐CoA ligase (GDP‐forming)	Involved in the TCA cycle

Note

Modified from Stumpf et al., 1985.

Abbreviations: ATP, adenosine triphosphate; GDP, guanosine diphosphate; TCA, tricarboxylic acid.

## 3 THE MECHANISM OF LEG CRAMPING IN PATIENTS UNDERGOING HEMODIALYSIS

Metabolic alkalosis is thought to be a key component of the etiology of the leg cramping that occurs in the latter half of hemodialysis sessions, but there have been conflicting reports of its exact role. Some previous studies have shown that metabolic alkalosis induces hypotension but does not affect the incidence of convulsions (Gabutti et al., 2003), and others have shown that it increases neuromuscular excitability and reduces cerebral blood flow, resulting in paresthesia, spasms, and convulsions (Ramin et al., 2006).

### 3.1 Alkalosis is the trigger for leg cramping

In general, muscle spasms occur because of the release of calcium ions from the sarcoplasmic reticulum. Plasma alkalosis induces the binding of calcium ions to serum albumin molecules (Pedersen, 1972), resulting in hypocalcemia. In addition, alkalosis induces the release of calcium ions from the sarcoplasmic reticulum (Nakamaru & Schwartz, 1972). With respect to leg cramping during hemodialysis, electromyography has shown that the muscle spasms are of peripheral nerve origin, but in the presence of alkalosis, the excitability of neuromuscular junction is also high, which exacerbates the problem (Millis et al., 1987).

### 3.2 Role of contraction alkalosis in the pathology of leg cramping in patients undergoing hemodialysis

Leg cramping during the latter half of dialysis sessions is more likely to occur in patients who have had a large amount of water removed (Donauer et al., 2000; McGee, 1990) and experience a condition referred to as contraction alkalosis. In general, changes in the volume of the extracellular fluid (ECF) are not reflected in the total amount of HCO_3_^−^ in the ECF. Therefore, if too much water is removed during dialysis, such that the ECF volume decreases, the concentration of HCO_3_^−^ increases (Garella et al., 1975; Haskins et al., 2006).

In patients undergoing maintenance hemodialysis, no significant changes in blood pH, pCO_2_, or the sodium or chloride concentrations after 1 L of water is removed by extracorporeal ultrafiltration. However, the HCO_3_^−^ concentration significantly increases from 21.15 ± 0.54 mmol/L to 23.05 ± 1.10 mmol/L (mean increase of 1.90 mmol/L) and the ionized calcium concentration tends to decrease from 1.91 ± 0.29 mmol/L to 1.68 ± 0.33 mmol/L (Takahashi et al., 2002).

The presence of sodium bicarbonate in the dialysate tends to cause alkalosis, and this is exacerbated by contraction alkalosis, such that muscle spasms are more common in patients from whom a large amount of water has been removed (Mujais, 1994). Furthermore, when an amount of water is removed that takes the patient below their target body mass, their blood pressure is often low, but leg cramping occurs because of excessive contraction alkalosis (Mujais, 1994).

## 4 THE EFFECTS OF CARNITINE DEFICIENCY

### 4.1 Carnitine deficiency during hemodialysis

Patients undergoing dialysis are known to be a risk of carnitine deficiency. The prevalence of carnitine deficiency has been reported to be 86.7% (Hatanaka et al., 2019) in patients undergoing hemodialysis and 82.3% (Shimizu et al., 2019) in patients undergoing peritoneal dialysis. This is the result of insufficient dietary carnitine intake, secondary to protein restriction (Borum & Taggart, 1986; Evans, 2003). Carnitine is synthesized primarily in the liver, kidneys, and brain from lysine and methionine, and this requires vitamin C, iron, vitamin B6, and niacin, (Rebouche, 1982; Rebouche, 2014) which are also deficient in patients undergoing dialysis (Descombes et al., 1993). In addition, the molecular weight of carnitine is 161.2 kg/kmol, which is almost the same as that of uric acid; therefore, 70%–80% of carnitine is removed from the blood during each hemodialysis session (Evans, 2003).

### 4.2 The direct effects of carnitine deficiency in muscle

The muscle‐related symptoms exhibited by patients undergoing hemodialysis include muscle cramps, muscle weakness, muscle fatigue, and myalgia. Mitochondrial dysfunction can substantially impair muscle function, as illustrated by the results of studies performed in mitochondrial DNA mutator mice (Yamada et al., 2012). A carnitine deficiency can result in similar defects that are referable to the mitochondria.

**FIGURE 3:**
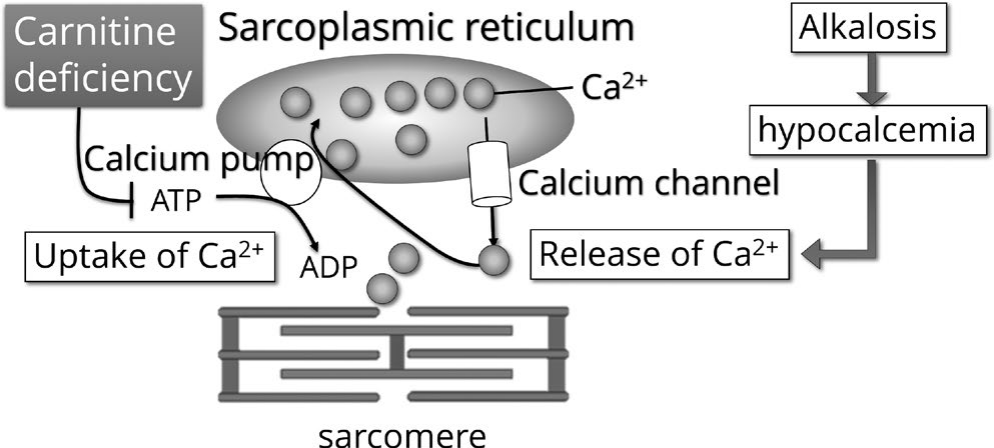
Controlling muscle spasms with carnitine. Alkalosis is involved in the etiology of muscle spasms, and contraction alkalosis is even more relevant for the leg cramping that occurs during the later stages of hemodialysis sessions. In general, alkalosis induces the binding of calcium ions to serum albumin, and therefore alkalosis causes hypocalcemia. Additionally, alkalosis makes it easier for calcium ions to be released from the sarcoplasmic reticulum of muscle cells, which leads to muscle cramping. The calcium pump on the sarcoplasmic reticulum consumes ATP and quickly reabsorbs the released calcium ions; therefore, muscle contractions are usually of short duration. However, in carnitine deficiency, muscle contractions are prolonged because of ATP depletion

Muscle contraction involves the release of calcium ions from the sarcoplasmic reticulum, such that the cytosolic calcium concentration rises 100‐fold, causing an interaction between actin and myosin (Ebashi, 1960). Normal contractions only last 5–10 ms, because ATP is consumed and the sarcoplasmic reticulum reabsorbs the calcium, rapidly reducing its cytosolic concentration. This is mediated by a calcium pump on the sarcoplasmic reticulum that transports two calcium ions for every ATP molecule used. However, carnitine deficiency results in ATP depletion without calcium reuptake into the sarcoplasmic reticulum, such that muscle contraction is maintained. To prevent muscle spasm, calcium release from the sarcoplasmic reticulum should be suppressed and sufficient ATP should be supplied to facilitate calcium reuptake into the sarcoplasmic reticulum (Figure 3). This effect can be achieved by ensuring the supply of sufficient carnitine.

When there is a deficiency of free carnitine, energy production is low and the concentration of acyl group‐containing molecules that are harmful to mitochondria is high (Sakurauchi et al., 1998). The importance of carnitine for long‐chain fatty acid oxidation is illustrated by the effects of systemic carnitine deficiency in lipid storage myopathy, and the etiology of myalgia includes defective energy production because of free carnitine deficiency and the presence of large numbers of molecules with acyl groups (Bonomini et al., 2011; Stephens et al., 2007).

It is thought that long‐chain acylcarnitines are not readily removed by hemodialysis, because of their hydrophobicity, micelle formation, or protein binding (Kamei et al., 2018). However, carnitine supplementation would be expected to promote the extracellular removal of excess mitochondrial acyl‐CoA as acylcarnitine, which should improve cellular homeostasis.

### 4.3 The central effects of carnitine deficiency

Muscle weakness and fatigue may also be secondary to central nervous system defects. Excess acyl groups are eliminated from cells by carnitine, and acetyl groups formed in muscles by transacetylation are also transported by carnitine. The resulting acetylcarnitine can pass through the blood–brain barrier and can be used to generate γ‐aminobutyric acid in the brain. Patients with chronic fatigue syndrome (CFS) have lower acetylcarnitine uptake in several regions of the brain (prefrontal and temporal cortices, anterior cingulate, and cerebellum), along with lower serum total carnitine, free carnitine, and acylcarnitine concentrations, in addition to lower acylcarnitine concentration (Plioplys & Plioplys, 1995).

Impaired transport of carnitine in the brain may also be the result of a physical defect in transport. A high serum concentration of transforming growth factor (TGF)‐β has been reported to be present in the majority of patients with CFS (Bennett et al., 1997), and TGF‐β is known to inhibit the production of dehydroepiandrosterone sulfate (Stankovic et al., 1994), which positively regulates the activity of carnitine acetyltransferase, the enzyme that catalyzes the transfer of free carnitine to acylcarnitine, and especially acetylcarnitine (Chiu et al., 1997).

Thus, carnitine deficiency may cause muscle weakness that is referable to the central nervous system, because carnitine may be involved in the sensitivity of the central nervous system to fatigue (Kuratsune et al., 2002).

## 5 THE TREATMENT OR PREVENTION OF LEG CRAMPING

### 5.1 The prevention of excessive contraction alkalosis and the reduction of overall alkalosis

To prevent excessive alkalosis, and therefore leg cramping, the composition of the dialysate and the method of dialysis should be adjusted to avoid overcorrection of acidosis. In addition, to prevent contraction alkalosis worsening the situation, it is important to limit both the patient's weight gain between dialysis sessions and the amount of water removed during dialysis (Van der Meulen et al., 1992).

If, despite these preventive measures, muscle spasm occurs during dialysis, the ECF should be rapidly increased, such that a dilution acidosis is induced to counteract the contraction alkalosis (Takahashi et al., 2002). A commonly used method to achieve this is the rapid injection of normal or hypertonic saline (Canzanello et al., 1991; Jenkins & Dreher, 1975). Shakuyakukanzoto (a Kampo, or traditional Chinese medicine), which contains kanzo (licorice), is also an effective means of relieving muscle cramps (Hinoshita et al., 2003), because licorice encourages water retention and dilution acidosis. However, this treatment must be used with caution, because excessive use of licorice results in pseudohyperaldosteronism and excessive fluid retention (Takeda et al., 1979). Furthermore, alkalosis can be reduced by treating hyperphosphatemia using sevelamer hydrochloride instead of calcium carbonate or lanthanum carbonate (Marco et al., 2002).

### 5.2 The use of carnitine for the prevention and treatment of muscle cramping

Carnitine supplementation is widely performed in patients undergoing hemodialysis because this is thought to ameliorate the muscular symptoms, including muscle weakness, muscle fatigue, myalgia, and cramping, that occur when carnitine is deficient, but this measure is not effective in every patient (Sakurauchi et al., 1998; Wanner & Hörl, 1988).

To be effective, a quantity of carnitine must be administered that provides sufficient ATP for the reuptake of calcium into the sarcoplasmic reticulum. Because approximately 80% of carnitine is removed during a hemodialysis session, date recommended ensuring a circulating concentration of 180 µmol/L before each hemodialysis session to achieve a normal circulating concentration of free carnitine (36–74 µmol/L). For a patient with a body mass of >50 kg, such a concentration can be maintained by the intravenous injection of 1 g L‐carnitine twice a week, and for a patient with a body mass of <50 kg, an intravenous injection of 1 g L‐carnitine once a week is sufficient (Date, 2020). This method of administration is currently being established in clinical practice in Japan.

Carnitine supplementation is performed in such patients because this is thought to ameliorate the muscular symptoms that occur when carnitine is deficient, but this measure is not effective in every patient (Wanner & Hörl, 1988). Sakurauchi et al., studied the effect of low‐dose L‐carnitine treatment (500 mg/d) on the muscle symptoms of 30 periodically dialyzed patients who demonstrated muscular weakness, fatigue, or cramps/aches during dialysis, and after 12 weeks of L‐carnitine treatment, approximately two‐thirds of the patients showed at least some improvement in their muscular symptoms (Sakurauchi et al., 1998). Furthermore, Takeuchi et al., reported that the number of patients who did not experience muscular symptoms significantly increased from 34.4% to 75.0% after 12 weeks of carnitine administration, and the percentage of patients in which muscle cramps appeared during each dialysis session decreased from 21.9% to 0% over the same time period. However, in another study, there was no correlation between the degree of improvement in symptoms after 24 weeks of carnitine administration and the increase in free carnitine concentration (Takeuchi et al., 2012). Thus, the effect of carnitine supplementation on muscle symptoms can be marked, but not all patients benefit equally.

### 5.3 The correction of other biochemical defects and nutrient deficiencies is required for carnitine administration to be effective

When attempting to increase ATP production in muscle, it should be noted that carnitine is involved in the process only as far as acyl‐CoA generation. The subsequent stages of β‐oxidation and turning of the TCA cycle require water‐soluble vitamins (vitamin B_1_, B_2_, B_6_, niacin, and pantothenic acid) (Niwa et al., 1975), which are easily removed during hemodialysis (Descombes et al., 1993). If the patient has a low alanine aminotransferase activity, vitamin B_6_ deficiency may be responsible (Chimata et al., 1994), and therefore, vitamin B_6_ and other water‐soluble vitamins should be prescribed. Furthermore, coenzyme Q10 (CoQ10, ubiquinone) is required for efficient operation of the electron transport chain and oxidative phosphorylation, but the administration of statins inhibits CoQ10 production, because the synthetic pathways for cholesterol and CoQ10 overlap (Marcoff & Thompson, 2007). Therefore, for patients in whom carnitine administration is ineffective, both water‐soluble vitamins and CoQ10 should be administered to ensure adequate ATP production, and if possible, statin administration should be discontinued. In addition, magnesium is necessary for proper operation of the TCA cycle, and magnesium ions suppress nerve and muscle excitability and are involved in neurotransmission and cell membrane stability (de Baaij, 2015; Varghese et al., 2020). Therefore, magnesium administration may also help to prevent leg cramping. Finally, choline is also removed by both hemodialysis (Ilcol et al., 2002a) and peritoneal dialysis (Ilcol, Dönmez, et al., 2002). Choline has been shown to inhibit the uptake of calcium ions into the sarcoplasmic reticulum in rabbit skeletal muscle (Beca et al., 2009) and choline deficiency affects muscle membrane lipid composition and intracellular lipid metabolism (Michel et al., 2011). On this basis, Moretti et al., suggested that choline plays important roles in energy production and cell membrane maintenance, and therefore its deficiency can also be a cause of muscle spasm and myalgia (Moretti et al., 2020). Furthermore, supplementation with choline significantly reduces creatine kinase activity in human patients (Fisher et al., 2007), and therefore choline administration may also be required in such patients.

## 6 CONCLUSION

To prevent or treat leg cramping, which often occurs during the second half of hemodialysis sessions, it is important to understand that the trigger is the acute onset of alkalosis and to determine whether this is caused primarily by the composition of the dialysate or excessive water removal, or by of ATP depletion as a result of a deficiency of carnitine and/or other nutrients. This will guide the choice of the most appropriate preventive measures, including carnitine administration.

## CONFLICT OF INTEREST

The author declares no conflict of interest.

## Data Availability

Data sharing is not applicable to this article, as no new data were created or analyzed during its preparation.
